# Aromatization Is Not Required for the Facilitation of Appetitive Sexual Behaviors in Ovariectomized Rats Treated With Estradiol and Testosterone

**DOI:** 10.3389/fnins.2019.00798

**Published:** 2019-08-06

**Authors:** Sherri Lee Jones, Stephanie Rosenbaum, James Gardner Gregory, James G. Pfaus

**Affiliations:** Department of Psychology, Center for Studies in Behavioral Neurobiology, Concordia University, Montreal, QC, Canada

**Keywords:** sexual desire, testosterone, estradiol, preclinical model, aromatase, fadrozole

## Abstract

Testosterone can be safely and effectively administered to estrogen-treated post-menopausal women experiencing hypoactive sexual desire. However, in the United States and Canada, although it is often administered off-label, testosterone co-administered with estradiol is not a federally approved treatment for sexual arousal/desire disorder, partly because its mechanism is poorly understood. One possible mechanism involves the aromatization of testosterone to estradiol. In an animal model, the administration of testosterone propionate (TP) given in combination with estradiol benzoate (EB) significantly increases sexually appetitive behaviors (i.e., solicitations and hops/darts) in ovariectomized (OVX) Long-Evans rats, compared to those treated with EB-alone. The goal of current study was to test whether blocking aromatization of testosterone to estradiol would disrupt the facilitation of sexual behaviors in OVX Long-Evans rats, and to determine group differences in Fos immunoreactivity within brain regions involved in sexual motivation and reward. Groups of sexually experienced OVX Long-Evans rats were treated with EB alone, EB+TP, or EB+TP and the aromatase inhibitor Fadrozole (EB+TP+FAD). Females treated with EB+TP+FAD displayed significantly more hops and darts, solicitations and lordosis magnitudes when compared to EB-alone females. Furthermore, TP, administered with or without FAD, induced the activation of Fos-immunoreactivity in brain areas implicated in sexual motivation and reward including the medial preoptic area, ventrolateral division of the ventromedial nucleus of the hypothalamus, the nucleus accumbens core, and the prefrontal cortex. These results suggest that aromatization may not be necessary for TP to enhance female sexual behavior and that EB+TP may act via androgenic pathways to increase the sensitivity of response to male-related cues, to induce female sexual desire.

## Introduction

The role of androgens and estrogens in male sexual behavior in rodent models has been well characterized (Hull et al., 1997; Sato et al., 2005; Hull and Dominguez, 2007), but the role of androgens given in combination with estradiol has not been well studied in female sexual behavior. This is particularly true for female sexually appetitive behaviors and the associated neural mechanisms, despite human data suggesting that testosterone plays a key role in female sexual desire. Testosterone is an effective treatment for estrogen-treated post-menopausal women experiencing hypoactive sexual desire (Sherwin et al., 1985; Burger et al., 1987; Shifren et al., 2000; Sherwin, 2002; Braunstein et al., 2005; Davis and Braunstein, 2012). However, one of the reasons that testosterone is not FDA-approved is due to a lack of understanding of the neural mechanisms through which it facilitates sexual desire in these women, and human studies cannot identify where in the brain testosterone is acting, nor the neural mechanisms through which it exerts its effects. Testosterone is an aromatizable androgen that can exert its effects through androgenic or estrogenic pathways and can activate androgen receptors directly or indirectly following conversion by 5α-reductase into dihydrotestosterone (DHT). Testosterone can also activate estrogen receptors following aromatization to estradiol (E2) (Simpson, 2002), or by increasing bioavailable E2 through the displacement of E2 from sex-steroid binding globulins, which bind androgens with higher affinity than estrogens (Burke and Anderson, 1972; Selby, 1990). Some early work has shown that both aromatizable and non-aromatizable androgens are involved in female rat sexual preference tests (de Jonge et al., 1986b), although comprehensive mechanistic studies of where in the brain and through which mechanisms testosterone facilitates sexual motivation when administered on an EB-baseline have not been conducted. Animal literature has identified hypothalamic, limbic, and the prefrontal cortex as key brain regions involved in the activation of female sexually appetitive behaviors, making them candidate regions where testosterone may exert its actions. Thus, a better understanding of the mechanisms through which testosterone facilitates sexual desire, in candidate brain regions, can be addressed using preclinical rodent models.

Animal studies have demonstrated that testosterone propionate (TP) can facilitate sexual behaviors in ovariectomized (OVX) as well as gonadally intact reproductively senescent female rats. Administration of TP to OVX rats treated with estradiol benzoate (EB) increases scent-marking frequency, proceptive behaviors and partner preference for sexually active males over EB administration alone (de Jonge et al., 1986a; Van de Poll et al., 1988). Administration of TP also synergistically increases proceptive (i.e., appetitive) sexual behaviors in OVX females treated with EB and progesterone (Fernández-Guasti et al., 1991), and to levels equivalent to treatment with EB and progesterone (Jones et al., 2017). It has also been shown that in the aged gonadally-intact female rat, TP capsules implanted subcutaneously acutely increase both appetitive and consummatory sexual behaviors (Jones et al., 2012). Recently it was reported that testosterone propionate (TP) administered to the sexually experienced EB-treated OVX Long-Evans rat, 4 h prior to testing facilitates appetitive sexual behaviors beyond the effect of EB alone (Jones et al., 2017). Thus, this rodent model can be useful for increasing our understanding of the mechanisms involved in TP-induced facilitation in an animal model of hypoactive sexual desire.

One potential mechanism through which testosterone can facilitate sexual desire is through an androgenic pathway. Testosterone binds to androgen receptors directly and indirectly following reduction to DHT, and numerous reports suggest this as a possible mechanism. Firstly, whereas estrogen replacement therapy alone does not restore decreased sexual function, desire and arousal in many postmenopausal women (Utian, 1975; Nathorst-Böös et al., 1993; Shifren et al., 1998), studies have shown that testosterone, even in the absence of E2, yields a modest, yet significant increase in sexual episodes and desire in post-menopausal women (Davis et al., 2008). Secondly, human studies have found a limited role of aromatization in testosterone’s ability to reinstate female sexual behavior. In one clinical study, post-menopausal women who were unresponsive to an estrogen therapy received transdermal testosterone in combination with either the aromatase inhibitor Letrozole, or placebo. Blocking aromatization with letrozole did not affect the enhancement in sexual satisfaction, general well-being and overall mood (Davis et al., 2006). In addition, Shifren et al. (2000) demonstrated that while a transdermal testosterone patch improved sexual function and well-being in postmenopausal women over placebo alone, serum free estradiol concentrations between these groups did not significantly differ, suggesting minimal aromatization. These results indicate that aromatization may not be necessary for testosterone to exert its facilitative role on female sexual desire in women treated with estrogens and suggest that facilitation may occur via an androgenic mechanism.

The first goal of the current study was to determine whether administration of the aromatase inhibitor fadrozole (FAD) would block the facilitation of female sexual behavior by TP in EB-treated females. Androgen receptors, estrogen receptors and the aromatase enzyme are widely distributed in the female brain, including the medial preoptic area (mPOA), ventromedial hypothalamus (VMH), and amygdala (Roselli et al., 1985, 1987; Handa et al., 1986, 1987; Wu and Gore, 2009; Wu et al., 2009; Feng et al., 2010; Stanić et al., 2014), and these are some key regions implicated in sexually appetitive behaviors. Thus, a second goal was to begin to address the activation of neural regions by testosterone’s facilitation of female sexual desire. To this end, we examined the number of Fos-immunoreactive (Fos-IR) cells within brain regions associated with sexual behavior (Pfaus and Heeb, 1997).

## Materials And Methods

### Animals

Sexually naive Long-Evans female rats (150–200 g), were obtained from Charles River (St-Constant, Quebec). Female rats were housed in pairs in shoebox cages in a reversed lighting schedule (12/12 h light-dark, with lights off at 8 p.m.). Food and water were given *ad libitum*. Male Long-Evans rats (200–250 g) obtained from the same supplier were used as stimulus animals (*n* = 33). These males were sexually experienced in the bi-level chambers with a group of OVX sexually experienced Long-Evans stimulus females primed with EB (10 μg/0.1 mL sesame oil) and progesterone (500 μg/0.1 mL sesame oil) administered 48 and 4 h prior to sexual training, respectively. Males were housed in groups of 3 or 4 in large plexiglass chambers lined with betachip. All other housing conditions were identical to those described for females.

All animal procedures were conducted in accordance with the standards established by the Canadian Council on Animal Care (CCAC) and approved by the Concordia University Animal Ethics Committee.

### Surgery

One week after arrival, experimental female rats were bilaterally ovariectomized (OVX) through lumbar incisions under a mixture of 4 parts ketamine hydrochloride to 3 parts xylazine hydrochloride administered by intraperitoneal injection (1 mL/kg of body weight). Females were treated post-operatively with subcutaneous injections of 3cc physiological saline, 0.03 mL Banamine and 0.1 mL Penicillin G.

### Hormone and Drug Preparation

All steroid compounds were received from Steraloids (Newport, RI). EB (10 μg), progesterone (500 μg), and TP (200 μg) were dissolved in 0.1 mL sesame oil under low heat for approximately 30 min, and stored at room temperature. Fadrozole hydrochloride (FAD; 1.25 mg/kg, Novartis Pharma and Sigma Aldrich) was dissolved in 0.1 mL of 0.9% physiological saline containing 20% 2-hydroxy propyl b-cyclodextrin and administered via subcutaneous injection twice a day (12 h apart). This dose was selected based on work showing that E2 was reduced in hypothalamic and amygdaloid nuclear pellets in FAD treated males compared to controls (Bonsall et al., 1992).

### Experimental Procedure

All sexual behavior training and testing occurred in bi-level chambers (Mendelson and Pfaus, 1989), during the middle third of the dark cycle. These chambers are designed to facilitate the experimenter’s view of the full behavioral repertoire of sexual behaviors (Mendelson and Pfaus, 1989; Pfaus et al., 1999). Males were placed in chamber alone for a 5 min habituation period. Next, females were introduced to the chamber for a 30 min training session.

After a 7 day post-operative recovery period, experimental females were primed with subcutaneous injections of EB 48 h before, and progesterone 4 h prior to each of four sex-training sessions with sexually vigorous males (Jones et al., 2013). The purpose of the sexual training sessions is to ensure that all females have sexual experience and to reduce variability in sexual responding (Gerall and Dunlap, 1973; and as in Jones et al., 2013). Following these 4 training sessions, females were given a 2 week hormone wash-out period before being randomly assigned to one of three experimental groups (*n* = 11/group). During this 2 week hormone wash-out, males were given 30 min training sessions with a different subset of sexually-experienced, hormonally-primed females every 4 days, to keep them sexually active.

**FIGURE 1:**
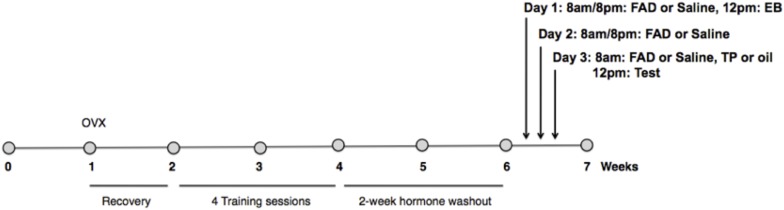
Experimental timeline. Females were ovariectomized 1 week after arrival into the colony, and given 1 week of recovery. All females were primed with estradiol benzoate (EB) 48 h before, and progesterone (P) 4 h prior to each of four sex-training sessions with males. After a 2 week hormone washout period females were randomly assigned to one of three experimental groups (*n* = 11/group): EB+Oil+Saline, EB+TP+Saline, or EB+TP+FAD (fadrozole). Estradiol benzoate (EB) was administered to experimental females by subcutaneous injection 48 h, and testosterone propionate (TP), or an equal volume of the oil control, 4 h before testing. FAD, or an equal volume of the vehicle control, was administered by subcutaneous injection at 8 a.m. and 8 p.m. every day for 3 days including the test day. For the experimental session, females were given 30 min to copulate with a sexually vigorous male.

EB was administered to experimental females by subcutaneous injection 48 h, and TP (or an equal volume of the oil control) 4 h before testing. FAD (or an equal volume of the vehicle control) was administered by subcutaneous injection at 8 a.m. and 8 p.m. every day for 3 days including the test day (Figure 1). For the experimental session, females were given 30 min to copulate with a sexually vigorous male.

All training and test sessions were video-recorded with a Sony Handycam, digital files were transferred to a personal computer, and sexual behaviors were scored blind to group condition using the Behavioral Observation Program (Cabilio, 1996) customized for rat sexual behavior.

### Behavioral Measures

Solicitations, defined as head-wise orientation toward the male followed by a run-away to the same or a different level, and hops and darts were used as measures of appetitive sexual behaviors (Pfaus et al., 1999; Jones et al., 2017). The consummatory measure, lordosis, was measured on a 4-point scale according to Hardy and Debold (1971) such that no lordosis was coded as a zero and increasing lordosis magnitudes (LM) from low to high were coded from 1 to 3. A lordosis quotient (LQ) was calculated by taking the ratio of total LMs to the number of mounts, intromissions and ejaculations received by the male. Mounts, intromissions and ejaculations received from the male were also coded (Pfaus et al., 1999).

### c-Fos Immunoreactivity

Two weeks following the test day, a subset of females that had been behavioral responsive on the test day (*n* = 5/group) were given their respective treatments of EB, EB+TP, or EB+TP+FAD, and were exposed to a sexually vigorous male behind a metal grid divider for 1 h prior to sacrifice. This was done so that females received only visual, auditory and olfactory cues from the males, since the goal was to investigate activation of regions involved in sexually appetitive behaviors without the confound of activation induced by receipt of sexual stimulation from the male, which is also known to differentially activate brain regions (Pfaus et al., 1993, 1994, 1996).

### Immunocytohistochemistry

Females were deeply anesthetized with an intraperitoneal injection of sodium pentobarbital (120 mg/kg/mL), and perfused intracardially with ice-cold phosphate-buffered saline (300 mL) followed by ice-cold 4% paraformaldehyde in 0.1 M phosphate buffer (300 mL). Brains were then removed, postfixed in 4% paraformaldehyde for 4 h, and stored overnight in 30% sucrose at 4°C.

### Histology

Frozen coronal brain sections were sliced using a cryostat from the olfactory bulb until the beginning of the cerebellum. All sections were rinsed in cold 0.9% 50 mM tris buffer saline (TBS) and put into a 30% hydrogen peroxide TBS solution and left for 30 min at room temperature. The sections were incubated for 2 h at room temperature in a 3% Normal Goat Serum (NGS) solution mixed in 0.2% triton TBS. Following the preblocking phase, sections were incubated for 72 h at 4°C in a solution containing: 3% NGS, primary rabbit polyclonal c-Fos antibody (Fos ab5, Calbiochem, Mississauga, ON; diluted 1:10,000) in a 0.05% triton TBS solution. Sections were transferred into a solution containing: 3% NGS, secondary antibody (Vector Laboratories Canada, Burlington, ON; 1:200) in a 0.2% triton TBS solution for 1 h at 4°C. Sections were then incubated for 2 h at 4°C in the avidin-biotinylated-peroxidase complex (Vectastain Elite, ABC kit, Vector Laboratories, diluted 1:55). Sections were washed in TBS (3 × 5 min) between each incubation. Sections were then washed for 10 min in a 50 mM Tris buffer solution (pH = 7.6) before transferring to 3,3′-diaminobenzidine (DAB) in 50 mM Tris (0.1 mL of DAB/Tris buffer, pH 7.6) for another 10 min. Finally, sections were incubated in a 8%NiCl_2_ (0.08 g) solution (400 μL per 100 mL of DAB/H_2_O_2_ solution). The DAB reaction was stopped by transferring the sections to cold TBS (3 ×10 min washes) at room temperature. Sections were then mounted on gel-coated slides and allowed at least 24 h to dry. Sections were then dehydrated for 10 min each in 70, 90, and 100% ethanols, and immersed in Xylene for 2 h. The sections were then coverslipped using permount glue and allowed to dry for 48 h before examination under a light microscope. Confirmation of successful Fos-IR was made when dark staining was detected within cell nuclei, as in Pfaus et al. (1993).

**FIGURE 2:**
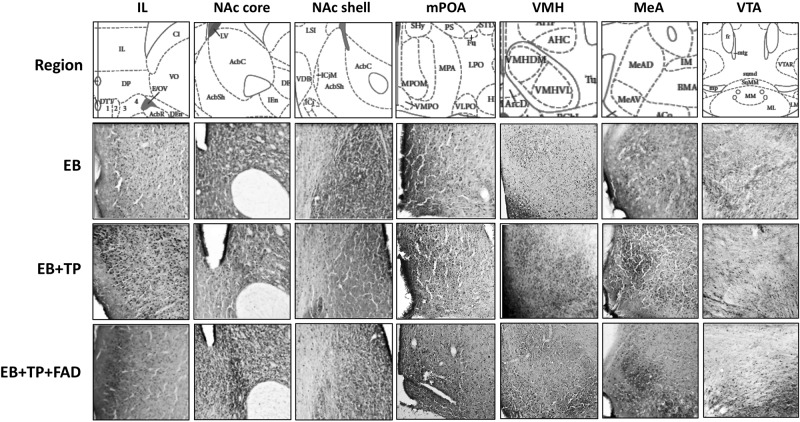
Representative pictures of Fos-immunoreactive cells taken at 40X magnification in different hypothalamic and limbic structures in ovariectomized female rats treated with estradiol benzoate (EB) alone, or in combination with testosterone propionate (TP), or TP and the aromatase inhibitor fadrozole (FAD). Landmarks used to identify regions of interest include the claustrum (cl) and the forceps minor corpus callossum just superior to the cl for the IL; the ventral extent of the lateral ventricle and the anterior portion of the anterior commissure (aca) for the NAc; the 3rd ventricle, optic chiasm and continuous anterior commissure for the mPOA; the 3rd ventricle and arcuate nucleus (Arc) and the three distinct VMH subdivisions for VMH sections; the optic tract (opt), internal capsule (ic), and piriform cortex for MeA sections, and the dorsal 3rd ventricle, medial mammillary nuclei (ML and MM, lateral and medial, respectively) and the fasciculus retroflexus (fr) for the VTA. IL, Infralimbic prefrontal cortex; NAc, Nucleus Accumbens; mPOA, medial preoptic area; VMH, ventromedial hypothalamus; MeA, medial amygdala; VTA, ventral tegmental area.

Tissue sections were examined at 40× and average numbers of Fos-IR cells were counted bilaterally using five sections for each region/rat, which appeared to contain the largest number of Fos-IR cells (Pfaus et al., 1993, 1996; Coria-Avila and Pfaus, 2007; Parada et al., 2010). Using the Paxinos and Watson (1986) rat brain atlas regions of interest were identified using standard visible anatomical landmarks (Pfaus et al., 1993, 1996; Smith et al., 1997; Coria-Avila and Pfaus, 2007; Parada et al., 2010). Fos-IR cells were counted in the infralimbic prefrontal cortex (IL; Plates 8–10), medial amygdala (MeA: Plates 27–29), medial preoptic area (mPOA: Plates 20–22), ventromedial hypothalamic nucleus (VMH; Plates 27–29) ventral tegmental nucleus (VTA: Plates 39–43), nucleus accumbens (NAc) core, and shell (Plates 11–15). The methodology applied for taking pictures, selecting the region of interest, and counting Fos-IR cells was as in previous papers from our group, but specifically, we applied methodology and regions of interest as previously reported in Coria-Avila and Pfaus (2007), Parada et al. (2010), Pfaus et al. (1993, 1996) and Smith et al. (1997). All pictures were taken by a researcher (JGG) blind to experimental group. The researcher identified and captured all sections containing the region of interest which could be identified with the visible landmarks (as described in Figure 2).

Images of each section were captured on a desktop computer under the same light intensity using Q Capture Pro (version 5.1) connected to a Leitz microscope (40×) and saved in TIFF format before importing into Image J. ImageJ software was used to count the number of Fos-IR cells in each region by a researcher blind to experimental group (SR). For each brain region, the region of interest was identified according to standard anatomical landmarks (Figure 2), then manually outlined on the sections containing the largest number of Fos-IR positive cells. It should be noted that this can lead to some minimal degree of inter-subject variability in the exact location of Fos-IR counts within the region of interest. The region of interest was identified and outlined as described in previous publications (Pfaus et al., 1993, 1996; Smith et al., 1997; Coria-Avila and Pfaus, 2007; Parada et al., 2010). Our methodology for counting Fos-IR cells consisted first, of adjusting the brightness and contrast on the first section counted using ImageJ and noting that contrast value to apply it to all subsequent images for that region. Next, the threshold tool was used to manually capture all cells that were subjectively identified as immunopositive, blind to experimental group. For all images, circularity was set to 0.3–1, and pixel size was set to 2–40.

### Statistical Analyses

Data were analyzed with Statistical Package for the Social Sciences (SPSS) software (Version 18). Due to violation of homogeneity of variance, the Kruskall–Wallis test was used to analyze behavioral differences between groups. *Post hoc* analyses were conducted using the Mann–Whitney U and a Bonferroni correction was applied for the three group comparisons (p_*adj*_), but unadjusted *p*-values are also reported for transparency and interpreted as trends. Effect sizes were computed on the Mann–Whitney tests using the formula *r* = Z/(sqrt(n)). The level of significance was set to 0.05 for all tests.

Data are presented using boxplots, and outliers were defined as generated by SPSS (outliers are defined as 1.5–3 times the interquartile range, and extreme outliers are defined as values 3 or more times the interquartile range). The number of animals that displayed at least one occurrence of the behavior was calculated. All animals were included in all analyses, except for lordosis measures, where only females that received a mount from a male were included, because the calculation of LQ and LM depends on mounts received.

Brain data were analyzed using a one-way analysis of variance (ANOVA) to test for differences between EB-alone, EB+TP and EB+TP+FAD groups, and significant ANOVAs were followed up with Fisher’s Least Significant Difference *post hoc* analysis. The level of significance was set at 0.05 for all comparisons. Eta square is reported as a measure of effect size for ANOVAs and Hedge’s g for between group comparisons.

## Results

The percentage of females displaying each behavior within each treatment group is shown in Table 1.

**TABLE 1:** Percentage of females (n) displaying sexual behaviors according to hormone treatment group (*N* = 11/group).

	**EB+O**	**EB+TP**	**EB+TP+FAD**
Hops and darts	45.5%(5)	81.8%(9)	72.7%(8)
Solicitations	0%	27.3%(3)	72.7%(8)
Level changes	100%(11)	100%(11)	100%(11)
Defensive behaviors	91%(10)	100%(11)	90.9%(10)
LQ^*a*^	0%	36.4%(4)	72.7%(8)
LR^*a*^	0%	36.4%(4)	72.7%(8)
Mounts	54.5%(6)	72.7%(8)	90.9%(10)
Intromissions	0%	18.2%(2)	72.7%(8)
Ejaculations	0%	9.1%(1)	63.7%(7)

**^*a*^For lordosis quotient (LQ) and lordosis rating (LR), only those females that were mounted could be included in the analyses. The number of females mounted (with or without intromission) per group are EB+O n = 6; EB+TP n = 8, EB+TP+FAD n = 11.**

### Appetitive Sexual Behaviors

**FIGURE 3:**
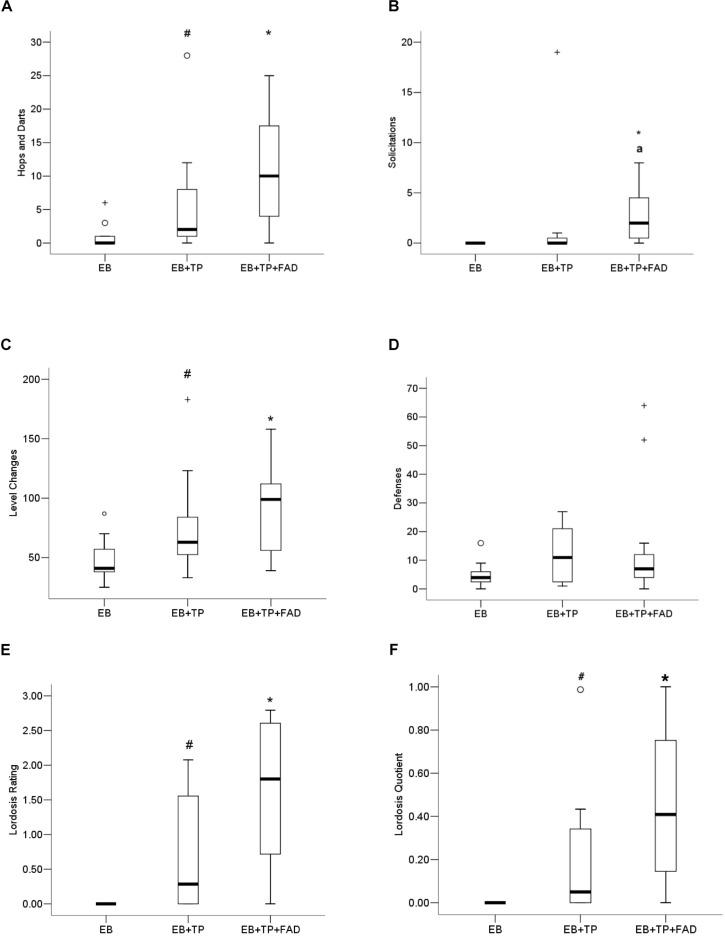
Median frequency of hops/darts **(A)**, solicitations **(B)**, level changes **(C)**, defensive behaviors **(D)**, lordosis rating **(E)**, and lordosis quotient **(F)** of ovariectomized Long-Evans rats (*n* = 11/group) treated with estradiol benzoate (EB) with or without testosterone propionate (TP) and the aromatase inhibitor fadrozole (FAD). Data were analyzed using Kruskall–Wallis to detect differences between groups, and significant effects were followed up using Mann–Whitney U, and *p*-values were adjusted using a Bonferroni correction. Boxes represent interquartile range, and whiskers each represent the top and bottom 25% of scores. ^*o*^ Outlier. +Extreme outlier. ^∗^Different from EB-alone, *p*_*adj*_ < 0.05. ^#^Tendency to differ from EB-alone, *p* < 0.05, or *p*_*adj*_ < 0.10. ^*a*^Tendency to differ from EB+TP, *p* < 0.05.

The non-parametric Kruskall–Wallis was conducted to test for behavioral differences between groups. Females treated with EB+TP+FAD displayed more hops/darts (Figure 3A) compared to EB-alone (*U* = 24, *z* = 2.486, *p* = 0.013, *p*_*adj*_ = 0.039, *r* = 0.53), and to levels equivalent to EB+TP (*U* = 44, *z* = 1.091, *p* = 0.275, *p*_*adj*_ = 0.825, *r* = 0.23; main effect, X^2^(2) = 7.530, *p* = 0.023), whereas EB+TP tended to increase the number of hops/darts compared to EB-alone (*U* = 30.5, 2.04, *p* = 0.041, *p*_*adj*_ = 0.123, *r* = 0.43). Sexual solicitations (Figure 3B) did not differ between females treated with EB+TP compared to EB-alone (*U* = 44, *p* = 0.069, *p*_*adj*_ = 0.207, *z* = 1.817, *r* = 0.39), whereas females administered EB+TP+FAD displayed significantly more solicitations compared to EB-alone (*U* = 16.4, *p* = 0.001, *p*_*adj*_ = 0.003, *z* = −3.354, *r* = 0.715) and tended to display more than females treated with EB+TP (*U* = 30, *p* = 0.032, *p*_*adj*_ = 0.096, *z* = −2.144, *r* = 0.46); main effect, X^2^(2) = 13.009, *p* = 0.001.

### Level Changes and Defensive Behaviors

More level changes (Figure 3C) were observed in females treated with EB+TP+FAD (*U* = 19, *p* = 0.006, *p*_*adj*_ = 0.018, *Z* = −2.727, *r* = 0.58) compared to those treated with EB-alone, whereas there was a tendency for EB+TP to increase level changes compared to EB-alone (*U* = 28, *p* = 0.033, *p*_*adj*_ = 0.099, *Z* = −2.137, *r* = 0.46); EB+TP and EB+TP+FAD did not differ (*U* = 47, *p* = 0.375, *p*_*adj*_ = 1.00, *Z* = −0.887, *r* = 0.19) [main effect, X^2^(2) = 8.625, *p* = 0.013]. Defensive behaviors (Figure 3D) did not differ between groups, X^2^(2) = 2.761, *p* = 0.251.

### Lordosis

Lordosis rating (LR; Figure 3E) was higher in females treated with EB+TP+FAD compared to EB-alone (*U* = 6.0, *p* = 0.005, *p*_*adj*_ = 0.015; *Z* = −2.781, *r* = 0.70) and tended to be higher in females treated with EB+TP compared to EB-alone (*U* = 9.0, *p* = 0.036, *p*_*adj*_ = 0.108, *Z* = −2.094, *r* = 0.45), whereas LR did not differ between females treated with EB+TP+FAD and EB+TP [*U* = 18.0, *p* = 0.093, *p*_*adj*_ = 0.279, *Z* = −1.680, *r* = 0.41; main effect, X^2^(2) = 9.455, *p* = 0.009]. LQ tended to be higher in females treated with EB+TP compared to EB-alone (*U* = 12, *p* = 0.052, *p*_*adj*_ = 0.156, *Z* = −1.940, *r* = 0.52, Figure 3F), and was significantly higher in females treated with EB+TP+FAD (*U* = 9.0, *p* = 0.009, *p*_*adj*_ = 0.027, *Z* = −2.612, *r* = 0.63) compared to EB-alone, whereas LQ did not differ between females treated with EB+TP and those treated with EB+TP+FAD [*U* = 28.0, *p* = 0.206, *p*_*adj*_ = 0.618, *Z* = −1.355, *r* = 0.31; main effect, X^2^(2) = 7.802, *p* = 0.020].

### Male Stimulations

**FIGURE 4:**
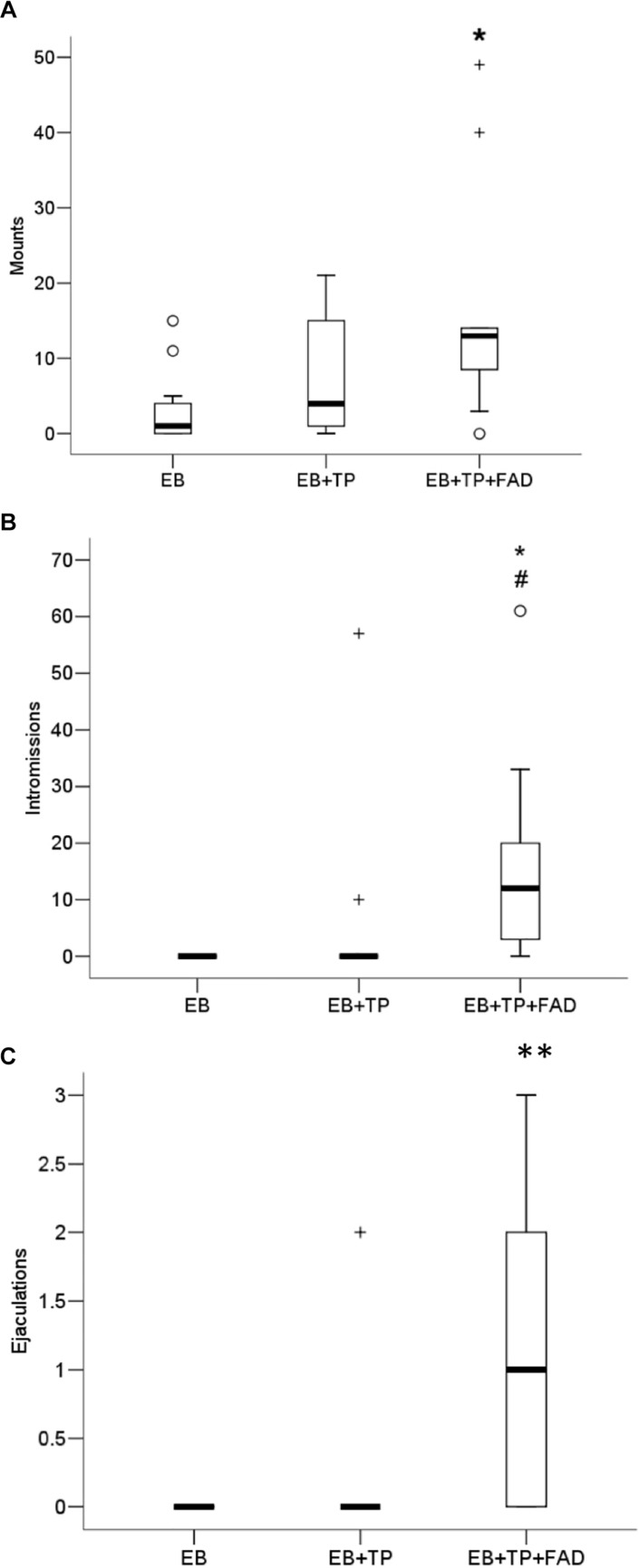
Median frequency of mounts **(A)**, intromissions **(B)**, and ejaculations **(C)** that males made toward ovariectomized Long-Evans rats (*n* = 11/group) treated with estradiol benzoate (EB) with or without testosterone propionate (TP) and the aromatase inhibitor fadrozole (FAD). Data were analyzed using Kruskall–Wallis to detect differences between groups, and significant effects were followed up using Mann–Whitney U, and *p*-values were adjusted using a Bonferroni correction. Boxes represent interquartile range, and whiskers each represent the top and bottom 25% of scores. ^*o*^ Outlier. ^+^Extreme outlier. ^∗^Different from EB-alone, *p*_*adj*_ < 0.05. ^#^Tendency to differ from EB-TP, *p* < 0.05; ^∗∗^Different from EB-alone and EB+TP, both *p*_*adj*_ < 0.05.

Females treated with EB+TP+FAD received significantly more mounts (Figure 4A) than females treated with EB-alone (*U* = 21, *p* = 0.008, *p*_*adj*_ = 0.024, *Z* = −2.626, *r* = 0.56), whereas females treated with EB+TP did not differ from EB-alone (*U* = 41.5, *p* = 0.200, *p*_*adj*_ = 0.600, *Z* = −1.281, *r* = 0.27), or from EB+TP+FAD [*U* = 38.5, *p* = 0.151, *p*_*adj*_ = 0.453, *Z* = −1.452, *r* = 0.31; main effect, X^2^(2) = 7.173, *p* = 0.028].

Whereas females treated with EB+TP did not differ from EB-alone in the number of intromissions received (*U* = 49.5, *p* = 0.148, *p*_*adj*_ = 0.444, *Z* = −1.447, *r* = 0.31), females treated with EB+TP+FAD received significantly more intromissions than females treated with EB-alone (*U* = 16.5, *p* = 0.001, *p*_*adj*_ = 0.003, *Z* = −3.353, *r* = 0.71) and tended to receive more than females treated with EB+TP [*U* = 28.0, *p* = 0.020, *p*_*adj*_ = 0.060, *Z* = −2.332, *r* = 0.50; Figure 4B; main effect, X^2^(2) = 13.729, *p* = 0.001].

Similarly, whereas females treated with EB+TP did not differ from EB-alone in the number of ejaculations received (*U* = 55.0, *p* = 0.317, *p*_*adj*_ = 0.951, *Z* = −1.000, *r* = 0.21), females treated with EB+TP+FAD received significantly more ejaculations than females treated with EB-alone (*U* = 22, *p* = 0.002, *p*_*adj*_ = 0.006, *Z* = −3.067, *r* = 0.65), and compared to females treated with EB+TP [*U* = 28.5, *p* = 0.014, *p*_*adj*_ = 0.042, *Z* = −2.451, *r* = 0.52; Figure 4C; main effect, X^2^(2) = 13.136, *p* = 0.001].

### Fos-IR

Descriptive data of all Fos-IR counts for each brain region by group are shown in Table 2, and representative pictures are shown in Figure 2. One-way ANOVAs were used to determine if there were significant differences between treatment groups, followed by an LSD *post hoc* analysis. EB+TP and EB+TP+FAD had higher Fos -IR counts than EB alone, in the mPOA [*p =* 0.023, *g* = 1.66; *p* = 0.01, *g* = 2.71, respectively, main effect of group, *F*_(2, 11)_ = 5.432, *p* = 0.023, *R*^2^ = 0.497], the NAc core [*p* = 0.02, *g* = 2.36, *p* = 0.01, *g* = 2.70, respectively, main effect of group, *F*_(2, 11)_ = 6.008, *p* = 0.022, *R*^2^ = 0.572], the IL [*p* = 0.005, *g* = 2.52, and *p* = 0.0048, *g* = 2.62, respectively, main effect of group, *F*_(2, 11)_ = 6.912, *p* = 0.015, *R*^2^ = 0.606], and the vlVMH [*p* = 0.024, *g* = 2.46; *p* = 0.022, *g* = 1.74, respectively, main effect of group, *F*_(2, 12)_ = 14.705, *p* = 0.036, *R*^2^ = 0.485] but EB+TP and EB+TP+FAD did not differ from each other (mPOA, *p* = 0.624, NAc core, *p* = 0.654; IL, *p* = 0.180, vlVMH, *p* = 0.850). In the VTA, EB+TP+FAD females had higher Fos-IR counts than EB-alone (*p* = 0.007, *g* = 2.95) whereas EB+TP tended to increase the number of Fos-IR counts compared to EB-alone (*p* = 0.08, *g* = 1.57), but EB+TP and EB+TP+FAD did not differ (*p* = 0.122) [main effect of group, *F*_(2, 10)_ = 6.409, *p* = 0.022, *R*^2^ = 0.616].

**TABLE 2:** Average ± SEM numbers of Fos-immunoreactive cells in different hypothalamic and limbic structures in ovariectomized female rats treated with estradiol benzoate (EB) alone, or in combination with testosterone propionate (TP), or TP and the aromatase inhibitor fadrozole (FAD).

**Region**	**EB**	**EB+TP**	**EB+TP+FAD**
MeA	125.49 ± 38.85	159.19 ± 29.5	169.50 ± 31.14
mPOA	72.77 ± 26.28	224.13 ± 50.06^∗^	251.03 ± 33.33^∗^
**NAc**			
Core	74.23 ± 6.18	161.77 ± 19.98^∗^	174.46 ± 23.6^∗^
Shell	273.37 ± 56.05	397.56 ± 47.85	402.33 ± 34.91
IL	83.40 ± 4.67	163.80 ± 17.26^∗^	134.92 ± 12.25
**VMH**			
Dorsomedial	19.81 ± 3.12	32.54 ± 5.92	27.79 ± 3.52
Ventrolateral	23.21 ± 2.46	38.81 ± 3.22^∗^	39.94 ± 6.36^∗^
VTA	58.83 ± 4.62	76.81 ± 6.62	91.36 ± 6.32^∗^

**n = 5/group. Some tissue sections were damaged or had poor staining, resulting from 3 to 5 sections per region. EB, Estradiol Benzoate; TP, Testosterone Propionate; FAD, Fadrozole Hydrochloride; MeA, Medial Amygdala; mPOA, Medial Preoptic Area; NAc, Nucleus Accumbens; IL, Infralimbic Prefrontal Cortex; VMH, Ventromedial nucleus of the hypothalamus; VTA, Ventral Tegmental Area. ^∗^Different from EB, p < 0.05.**

No differences between groups were found in the dmVMH, *F*_(2, 1__2__)_ = 1.874, *p* = 0.204, *R*^2^ = 0.273, the NAc shell [*F*_(2, 11)_ = 2.041, *p* = 0.186, *R*^2^ = 0.312], or the MeA [*F*_(2, 12)_ = 0.455, *p* = 0.647, *R*^2^ = 0.083].

## Discussion

The purpose of this study was to determine whether administration of the aromatase inhibitor FAD would disrupt the facilitation of female sexually appetitive behaviors that occurs with TP treatment in EB-treated OVX rats, and to determine whether Fos-IR differed between groups in brain regions known to be involved in sexual motivation and reward. The present results illustrate that blocking aromatization using FAD in females treated with EB+TP increased hops/darts, solicitations, level changes and lordosis measures compared to those treated with EB-alone. These findings suggest that aromatization of TP to estradiol is not necessary for the display of female sexual behaviors in OVX rats treated with EB and TP. The Fos-IR data suggest that TP may act within the mPOA, NAc core, IL, and vlVMH to elicit its effects, and as well as the VTA, which specifically had higher numbers of Fos-IR cells in the EB+TP+FAD group compared to the EB-alone group.

In the current study, the behavioral levels induced by EB+TP were less pronounced than levels reported in Jones et al. (2017), particularly for LQ. However, this is not surprising given previous reports that a number of factors can influence behavioral sensitivity to estradiol, such as sexual experience (Gerall and Dunlap, 1973; Pfaus et al., 1999), EB dose (Pfaus et al., 1999; Jones et al., 2013), strain (Jones et al., 2013), bedding type (Jones et al., 2015), and exposure to male cues (Jones et al., 2015). Important individual differences exist in behavioral sensitivity to hormone treatments on sexual behavior. Thus, to ensure that females were all behaviorally sensitive to sex steroid hormones, we examined behaviors induced by EB+P priming on the fourth day of behavioral training, and for all groups LQ and LR were near maximal (range LQ = 0.93–0.98; range LR = 2.43–2.69), and no differences were detected between groups on any behavioral measure (Supplementary Table 1) suggesting that on average, the groups were equally as responsive to sex steroid hormones under equivalent and optimal hormone priming conditions. The variability in sensitivity to EB and TP is reminiscent of reports in the human literature, showing that some women’s low sexual desire responds rather well to estrogens administered alone, and that testosterone can be particularly beneficial to improving sexual desire in women who are unresponsive to estradiol alone, as originally reported by Burger et al. (1987). In addition to the environmental and experiential factors outlined above, hormone sensitivity can be dependent on differences in biological mechanisms, such as steroid hormone receptor density, enzymes, and hormone binding globulins, among other factors. Although the effectiveness of surgical ovariectomy and hormone administration were not formally tested, the high and normal levels of behavioral responding during the training phase, as well as the low level of responding in the control groups suggest that those manipulations were effective. Additional research will be needed to increase our understanding of individual differences in hormone sensitivity, and to determine who responds best to which treatments. Such considerations are already being taken into account for women presenting with differing etiologies (i.e., top-down or bottom-up sexual inhibition) of hypoactive sexual desire (e.g., Sarin et al., 2013; Poels et al., 2014).

The facilitation of EB+TP compared to EB-alone did not attain the strict statistical cut-offs in the current study, in contrast to the statistically significant increase in appetitive behaviors reported in Jones et al. (2017). We note however that the pattern of results in the current study mimic those reported in Jones et al. (2017), and moreover, the effect sizes on appetitive behaviors between EB-alone and EB+TP treated animals are similar in magnitude in the current study (hops/darts *r* = 0.43; solicitations *r* = 0.39; level changes *r* = 0.46) and Jones et al. (2017) (hops/darts *r* = 0.68, solicitations *r* = 0.68, level changes *r* = 0.60). The effect sizes range from moderate to large, suggesting a reliable and moderate ability for TP to facilitate appetitive sexual behaviors in EB-treated OVX female rats.

In the present study, blocking aromatase in OVX EB+TP treated rats enhanced appetitive sexual behaviors beyond that of EB-alone. The administration of TP tended to increase hops/darts and level changes beyond that of EB-alone, with moderate effect sizes on hops/darts, level changes, as well as solicitations (with *r* ranging from 0.39 to 0.46). The administration of FAD to females treated with EB+TP enhanced appetitive measures of sexual behaviors, such that EB+TP+FAD displayed significantly more hops/darts than EB-alone, and tended to display more sexual solicitations than females treated with EB+TP. The effect sizes between EB and EB+TP+FAD were moderate to large, with *r* = 0.53 for hops/darts and *r* = 0.715 for solicitations, and small to moderate between EB+TP and EB+TP+FAD, with *r* = 0.23 for hops/darts and *r* = 0.39 for solicitations. These findings suggest that aromatization to estradiol is not necessary for the facilitation of appetitive sexual behaviors by TP when administered to EB-treated females.

One strict interpretation of the present data is that FAD had no statistically significant facilitative effect on appetitive sexual behaviors beyond treatment with EB+TP (i.e., only a statistical trend for FAD to increase solicitations beyond EB+TP was detected). This interpretation could suggest that FAD may release an inhibitory effect induced by EB+TP (given that EB+TP+FAD facilitated sexually appetitive behaviors beyond EB-alone), which could involve, for example, extragonadal estradiol synthesis. However, an inhibitory action of extragonadal estradiol in the context of these data may not be a likely explanation for two reasons. First, estradiol is not inhibitory to sexual behavior in OVX oil-treated animals, and is necessary for the display of sexual behaviors (Pfaff, 1980). One mechanism through which EB+TP is thought to exert its effects is by indirectly increasing bioavailable estradiol, following its displacement from steroid hormone binding globulins by testosterone (Burke and Anderson, 1972). However, in our OVX females, endogenous levels of estradiol are probably too low, even given the multiple sites of extra-gonadal synthesis of estradiol (Barakat et al., 2016), particularly because FAD was administered twice a day for the duration of the experimental phase, and has previously been shown to effectively reduce E2 in hypothalamic nuclear pellets (Bonsall et al., 1992). As such, a more likely explanation is that more free androgen was available to act on androgen receptors to facilitate sexual behaviors. Second, when considered with previous publications using similar methods, EB+TP facilitates appetitive sexual behaviors beyond EB-alone, as discussed above. Nonetheless, we cannot rule out the interpretation that TP+FAD releases inhibition in EB-alone treated OVX females, particularly given that FAD was administered systemically, and that we did not measure circulating E2, nor did we confirm that FAD effectively reduced neural estradiol in our animals.

A more plausible and parsimonious interpretation of these data, particularly when considered in the context of the data presented in Jones et al. (2017) is that TP given in combination with EB facilitates appetitive sexual behaviors, at least in part, through androgenic mechanisms (Cappelletti and Wallen, 2016). As discussed above, TP induces a reliable and moderate increase in appetitive behaviors in EB-treated females, which was not blocked by FAD administration. This is consistent with previous results indicating the importance of androgen receptor activation in female sexual behavior (Jones et al., 2010; Kudwa et al., 2010). Testosterone has been shown to require the presence of estradiol to exert its modulatory role on female sexual behavior (Sherwin and Gelfand, 1987; Buster et al., 2005), thus it is possible that EB administration 48 h before testing upregulates androgen receptors, thereby facilitating the ability of testosterone to act on androgen receptors in areas of sexual behavior as it does with progesterone (Rubin and Barfield, 1983). It is also interesting that EB+TP+FAD tended to enhance the expression of sexual solicitations beyond that of EB+TP, and the only brain region that revealed increased Fos-IR specifically in the EB+TP+FAD group was the VTA. The VTA, a core component of the mesocorticolimbic reward pathway, contains androgen receptors (Kritzer, 1997; Kritzer and Creutz, 2008) and therefore this is a key region of interest for future mechanistic studies.

Some earlier animal studies have shown the importance of AR in the facilitation of female sexual behavior. For example, Yahr and Gerling (1978) demonstrated that administration of 6-alpha-fluorotestosterone, a non-aromatizable androgen, could induce sexual receptivity in female rats comparable to that of TP. In addition, recent studies using selective androgen receptor modulators (SARM) have revealed an important role of ARs in female sexual behavior. Administration of a non-aromatizable SARM that does not interact with estrogen receptors, to OVX rats primed with sub-optimal levels of EB (2.0 μg) + progesterone (100 μg) increased both proceptive and receptive sexual behavior in sexually-experienced females (Kudwa et al., 2010). Moreover, TP given in combination with R-bicalutamide, an anti-androgen, reduced sexual preference of a female for an intact male compared to TP-alone (Jones et al., 2010). Together these data highlight the importance of ARs and contribute to a more mechanistic approach underlying testosterone’s role in female sexual behavior.

Additionally, treatment with FAD appears to have increased the female’s attractivity. EB+TP+FAD-treated females received more mounts and intromissions than EB-alone treated females and tended to receive more intromissions than EB-TP, and receive more ejaculations than both the EB-alone and EB+TP treated females, all with correspondingly moderate effect sizes. We suspect that the behavior of the males was influenced by the appetitive behaviors and receptivity of their female partners, which is also reflected in the percentage of females that were mounted (i.e., about half the EB-treated females, and 73% of the EB+TP, and 91% of the EB+TP+FAD females). Pfaus and Pinel (1989) demonstrated that when training a male with a non-receptive female, the male quickly learns that she is not receptive followed by a drastic decrease in rate of mounting over trials. In the present study, the male’s mounts, intromissions and ejaculations on the final training day, occurring 2 weeks prior to testing were normally distributed, and 100% of females in each group were mounted (see Supplementary Table 1). Therefore, the males’ sub-par sexual behaviors toward females receiving EB-alone and EB+TP could be explained by the low appetitive and receptive behaviors displayed by these females, a behavioral pattern consistent with our previous reports of OVX Long-Evans rats treated acutely with EB-alone (Jones et al., 2013, 2017).

As a first step to investigating potential brain regions where TP may be exerting its effects to facilitate sexual motivation, Fos-IR was examined within mesocorticolimbic regions known to be involved in sexual motivation (Pfaus, 2009) following EB treatment and exposure to a male behind a screen. Fos-IR was investigated within the mPOA, MeA, IL, VMH, VTA, and NAc core and shell. TP administration to OVX EB-treated females induced Fos-IR in the mPOA, NAc core, IL and the vlVMH, whereas activation within the VTA occurred with the addition of FAD. These regions have a moderate to high density of ARs (Handa et al., 1986, 1987; Fernández-Guasti et al., 2000; Wu et al., 2009; Feng et al., 2010), making them potential candidate regions where TP may exert its effects.

The mPOA is a critical component in mediating female proceptive behaviors such as hops, darts and solicitations (Erskine, 1989b; Hoshina et al., 1994), and is important for the integration and interpretation of olfactory and auditory sensory cues (Hull et al., 1997). In the current study we found that compared to EB-alone, Fos-IR was expressed in more cells in females treated with EB+TP regardless of whether FAD was administered. These Fos-IR data parallel the behavioral data, namely the higher appetitive measures compared to EB-alone. Activity in the mPOA is sensitive to changes in hormonal milieu, thus one possible mechanism is that TP is working in the female mPOA as it does in the male, to modulate the mPOA’s neural responsiveness to olfactory cues (Pfaff and Pfaffmann, 1969). TP has also been shown to upregulate nitric oxide synthase, which increases levels of nitric oxide, thereby increasing dopamine release in the mPOA of male rats (Lorrain et al., 1996; Hull et al., 1997; Hull and Dominguez, 2006). This relationship has not been examined in the female brain, and we acknowledge that the mechanisms may be different between the sexes. Nonetheless, it is a likely candidate mechanism given that dopamine and the activation of its distinct receptors (D1 and D2) in the mPOA has been shown to mediate female sexual behavior (Matuszewich et al., 2000; Graham and Pfaus, 2010, 2012). Therefore, within the mPOA, TP may act through androgenic mechanisms. Future mechanistic studies are needed to determine the combined effects of estrogens and androgens on female sexual motivation within the female mPOA.

Upstream of the mPOA, the amygdala is important for integrating sensory information from the environment. The MeA itself is involved in female sexual motivation, via dopaminergic and progesterone signaling (Holder et al., 2015). The lack of difference in Fos-IR expression within the MeA between groups suggests that testosterone does not act within this region to facilitate appetitive sexual behaviors, and further suggest that all the females were detecting similar sensory input in response to male cues.

The vlVMH is well-known as a critical region for the expression of lordosis via estradiol signaling (Pfaff, 1968; Pfaff and Sakuma, 1979; Pfaff et al., 2000, 2011). In the current study, the vlVMH had significantly more Fos-IR nuclei in females given either EB+TP or EB+TP+FAD, when compared with EB alone. Consistent with this, EB+TP females displayed higher LR and LQ compared to EB-alone. There is evidence that certain androgens, such as DHT and 5α-androstane-3α,17β-diol, inhibit EB-induced lordosis in female rats (Baum and Vreeburg, 1976; Erskine, 1989a), and as such it is somewhat surprising that FAD led to a significant increase in lordosis measures beyond EB-alone, given that FAD is an aromatase inhibitor, which suggests that TP acted via an androgenic pathway. In summary, it is unclear through what mechanism within the vlVMH EB+TP+FAD might facilitate lordosis, although downstream midbrain mechanisms cannot be ruled out (Pfaff, 1980).

The dopaminergic output from the mPOA to the VTA is essential for sexual behavior (Brackett and Edwards, 1984). Females receiving EB+TP+FAD had significantly more Fos-IR in the VTA compared to females receiving EB alone. Downstream of the VTA, EB+TP+FAD, and EB+TP had significantly higher Fos-IR expression in the NAc core, although not in the shell, when compared to EB alone. The NAc has been implicated in the motivation to engage in sexual behavior, as well as in the rewarding properties of sexual behavior such as paced mating in the female rat (Jenkins and Becker, 2001, 2003; Guarraci et al., 2002, 2004). Specifically, the NAc shell has been shown to be involved in processing of rewarding stimuli, while the core is involved in motor function related to reward and reinforcement (Ito and Hayen, 2011). Infusion of the testosterone metabolite 3a-diol into the NAc shell selectively increased appetitive sexual behaviors (hops darts and ear wiggles) (Sánchez Montoya et al., 2010). Because in the current study the administration of TP to OVX EB-treated females upregulated Fos-IR in the NAc core but not shell, and that occurred regardless of whether FAD was also administered, it is likely that the effect of TP within the NAc is associated with the rewarding properties of sexual stimuli, or with the rewarding properties of TP itself (Nyby, 2008). It should be noted, however, that the dose of FAD used in this study was selected based on work showing that estradiol was reduced in hypothalamic and amygdaloid nuclear pellets in FAD-treated male rats compared to controls (Bonsall et al., 1992). Thus, because we did not measure aromatase activity in our female animals following FAD administration, we cannot be certain that the dose had the same level of effectiveness as reported by Bonsall et al. (1992).

## Conclusion

In conclusion, administration of FAD enhanced the facilitation of appetitive and consummatory sexual behaviors in OVX female rats treated with EB and TP, showing that aromatization of testosterone to estradiol is not required for TP-induced facilitation of sexual desire in our preclinical model. Moreover, TP-induced activation of Fos-IR expression in brain areas implicated in sexual motivation, behavior and reward, suggests that TP may increase the sensitivity to male-related cues and may enhance the female’s attractivity to the male. Future mechanistic studies should investigate whether the facilitation by TP can be blocked by giving androgen receptor inhibitors, and measuring circulating levels of estradiol, testosterone, and SHBG to better inform the mechanisms.

## Ethics Statement

All animal procedures were conducted in accordance with the standards established by the Canadian Council on Animal Care (CCAC) and approved by the Concordia University Animal Ethics Committee.

## Author Contributions

SJ designed the experiments in consultation with JP, trained SR and JG on the methodological details of the experiments, performed the surgeries, oversaw all aspects of the experiments, ran the final statistical analyses, and contributed to the intellectual content of the manuscript, and prepared the manuscript for publication. SR conducted the experiments, including preparation of solutions, injections, perfusions, histology, scored the behavior and performed the Fos counts, managed the data sets, conducted the analyses in consultation with SJ and JP, and wrote the first draft of the manuscript. JG assisted in all phases of data collection and in particular the brain staining and picture taking, provided intellectual contributions to the manuscript, and data interpretation. JP conceived and designed the experiments in collaboration with SJ, oversaw all aspects of the experiments, and provided intellectual contributions to the manuscript. All authors approved all the contents of the manuscript for publication.

## Conflict of Interest Statement

JP is a member of the scientific advisory boards for AMAG Pharmaceuticals, Emotional Brain, LLB., IVIX Corp, and Palatin Technologies, Inc. The remaining authors declare that the research was conducted in the absence of any commercial or financial relationships that could be construed as a potential conflict of interest.
